# The Treatment of Neuralgic and Rheumatic Affections

**Published:** 1896-11

**Authors:** D. S. Maddox

**Affiliations:** Marion, Ohio


### THE TREATMENT OF NEURALGIC AND RHEUMATIC AFFECTIONS.

D. S. MADDOX, M. D., MARION, OHIO.

In spite of extensive researches into the functions of the nervous system, we have not yet succeeded in obtaining precise and certain data concerning neuralgia. Austie thus defines neuralgia: "A disease of the nervous system manifesting itself by pains which appear to follow the course of certain nerves, ramifying sometimes into a few, sometimes into all the terminal branches of those nerves." What is of importance for us to know from its bearing on treatment is the etiology and pathology of this affection. In order that the functions of the nervous system may be normally performed, two conditions must exist, viz.:

1. The integrity of the nervous system itself, its cells and fibres.
2. The integrity of the circulatory system.

Another affection whose primal cause is often a matter of as much doubt as is that of neuralgia, is chronic rheumatism. This is a term which is loosely applied to many ailments not really of rheumatic origin. Almost any obscure and obstinate pain which is not traceable to some other agency is apt to be attributed to chronic rheumatism. Under this head then, there come to be ranked many aches and ailments which, not being of rheumatic origin, have no claim to the title.

Chronic rheumatism, properly so-called, is a milder form of the subacute variety in which there is not sufficient local inflammation to prostrate the patient or to raise the temperature. Just as the acute runs into the subacute, so the subacute runs into the chronic by the insensible gradations. It also exists independently of them. The malady is characterized by the occurrence of pains obstinate in nature, and sometimes shifting in character, affecting the joints, muscles and fibrous capsules. The affected parts may be somewhat tender to the touch, but are not, as a rule, distinctly swollen. The pain is increased by damp and cold. It often disappears in fair and returns in wet weather. It is a troublesome ailment which frequently lasts off and on for months, even years. During its continuance there is often laid the foundation of future cardiac troubles. In the age, in the personal and family history of the patient, in the shifting character of the pains, and in the occasional slight rise of the temperature, we have the best means of distinguishing true chronic rheumatism from the other ailments, gouty, arthritic and neuralgic, with which it is often confounded. The treatment of neuralgic and rheumatic affections is both constitutional and local. For some time now I have been using the Tongaline preparations in the treatment of these maladies, and the results so far have been most gratifying.—*Extract from the September Number of the Medical Summary.*

